# Ecthyma gangrenosum and severe transitory neutropenia in an immunocompetent girl^☆^^☆☆^

**DOI:** 10.1016/j.abd.2020.03.009

**Published:** 2020-07-15

**Authors:** Pablo Vargas-Mora, Santiago García, Ligia Aranibar, Fernando Valenzuela

**Affiliations:** Department of Dermatology, Faculty of Medicine, University of Chile, Santiago, Chile

Dear Editor,

Ecthyma gangrenosum (EG) is a rare cutaneous lesion, principally caused by *Pseudomonas aeruginosa*, through either primary infection or hematogenous spread. Other pathogenic agents responsible have also been described, such as *Aeromonas hydrophila*, *Staphylococcus aureus*, and *Aspergillus* spp., among others.^1^, ^2^ It generally develops in patients with sepsis or immunosuppression, in a context of hematological malignancies or immunosuppressive therapy. It presents with erythematous/violaceous or hemorrhagic lesions that evolve into a central necrotic ulcer with an erythematous halo, preferentially situated on the buttocks and legs.^1^ This report presents the case of a previously healthy baby girl with genital EG and the subsequent development of a severe transitory neutropenia.

A previously healthy 17-month-old girl presented with a hemorrhagic blister on the left side of her vulva. It had started seven days before, with perilesional erythema (Fig. 1) that progressively developed ulceration and edema. There was no fever or other systemic symptoms. She was treated with oral cefpodoxime and clindamycin. As there was no improvement after 72 hours, she was hospitalized and referred to dermatology. On physical examination, the patient was in good general condition with stable hemodynamics and no fever. She had a skin ulcer on the left labia majora with a maximum diameter of 1.7 cm and well-defined borders. There was fibrin at its base, much swelling, and perilesional induration, sensitive to the touch (Fig. 2). A hemogram was carried out which showed 5,840 leucocytes/mm^3^, an absolute neutrophil count (ANC) of 876 mm^3^, and C-reactive protein of 33 mg/dL. Polymerase chain reaction (PCR) tests of the lesion for herpes simplex viruses 1 and 2, cytomegalovirus, varicella-zoster virus, and the Epstein-Barr virus were all negative. Blood, fungal, and mycobacteria cultures were all negative, but a culture of the lesion tested positive for *Pseudomonas aeruginosa*. An immunological study was made for lymphocytic sub-populations, IgA-IgM-IgG immunoglobulins, neutrophil oxidative burst test, anti-neutrophil antibodies, VDRL, and HIV. All these were negative, thus eliminating the possibility of associated immunodeficiency. After treatment with intravenous ceftazidime and amikacin for six days there was a favorable evolution, so it was decided to discharge her and provide oral ciprofloxacin for 14 days. This resulted in the complete resolution of the lesion. At an outpatient checkup 72 hours after the discharge, the hemogram showed 4,800 leucocytes/mm^3^ and an ANC of 96 mm^3^, which improved spontaneously after two weeks and showed no relapse after six months on follow-up.

**Figure 1:**
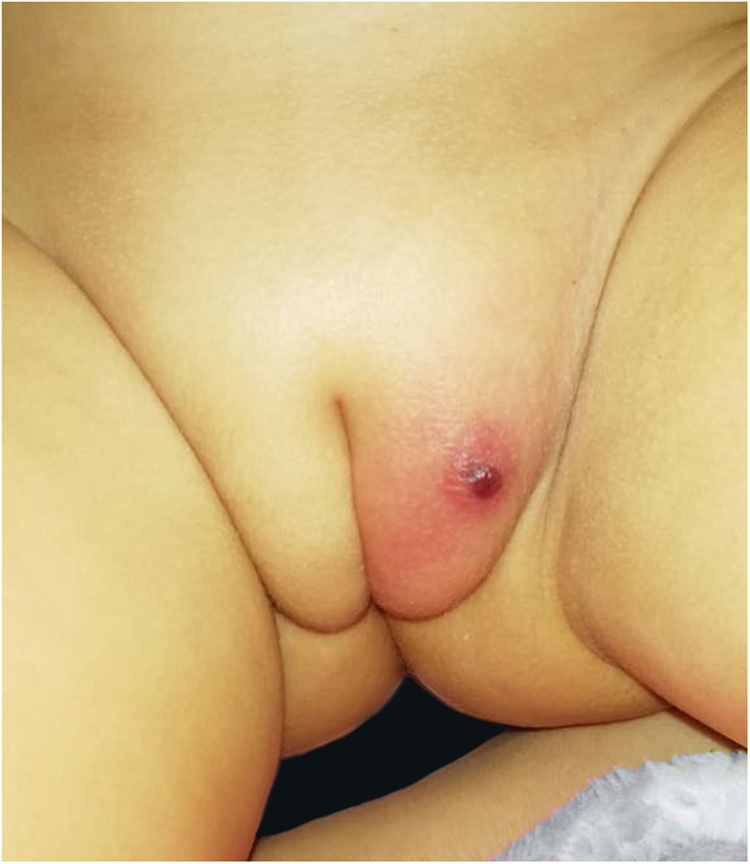
Hemorrhagic blister on vulva, two days of evolution.

**Figure 2:**
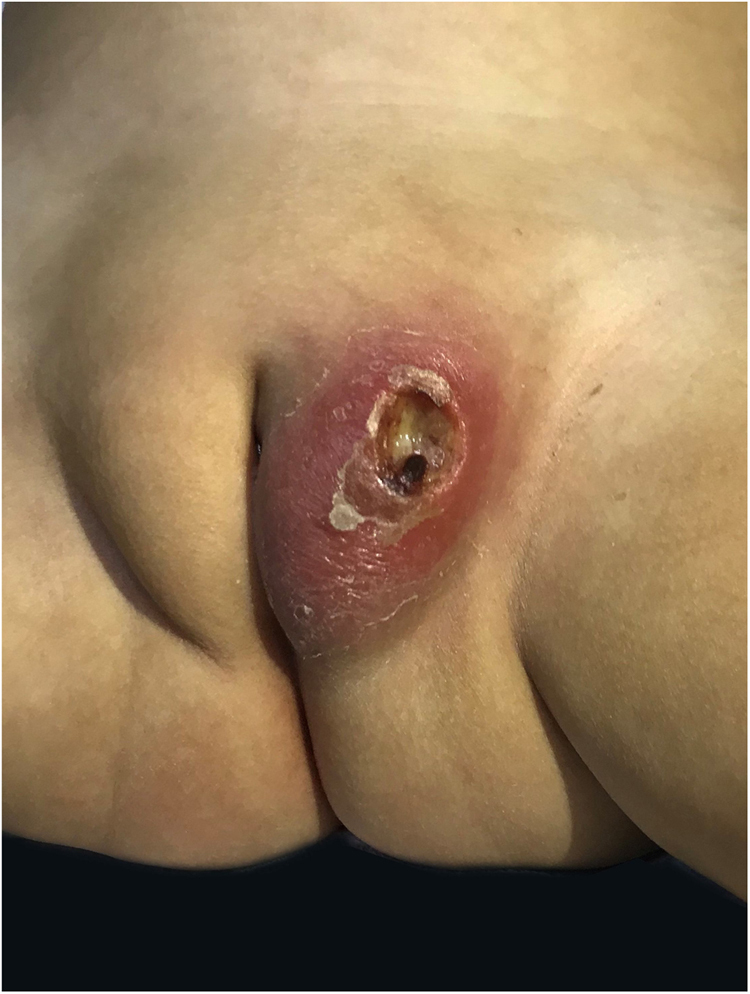
Skin ulcer in labia majora, seven days of evolution.

EG is a rare disease in healthy pediatric patients with no associated sepsis. It generally reveals a primary sub-clinical immunodeficiency and so it is essential to carry out a complete immunological study on all such patients. Its mortality is high, over 90% where there is sepsis and 15% in the case of local infection, where neutropenia is the most important factor for prognosis.^1^, ^3^, ^4^ In the literature there are few cases of EG in previously healthy patients; the present authors found only six which showed severe neutropenia (< 500 mm^3^) after the initiation of the clinical condition, as in this report. However, all these cases showed an associated etiological factor (respiratory infections, infantile benign neutropenia, and hypogammaglobulinemia).^4^, ^5^ Leucopenia induced by fluoroquinolones has been described. This tends to be mild to moderate, being reported in less than 0.2% of the cases, mainly in adults with co-morbidities.^5^ In the present case normalization of the hemogram occurred at the end of the second week of oral ciprofloxacin, so this cause is not very probable. Also, it has been suggested that *Pseudomonas aeruginosa* might cause a transitory neutropenia mediated by a toxin that could inhibit the migration of neutrophils to the affected areas and also reduce the number of neutrophils in the blood.^4^, ^5^

This case is reported due to the exceptional manifestation of severe neutropenia after EG in an immunocompetent patient.

## Financial support

None declared.

## Authors' contributions

Pablo Vargas-Mora: Approval of the final version of the manuscript; drafting and editing of the manuscript; intellectual participation in the propaedeutic and/or therapeutic conduct of the studied cases; critical review of the literature; critical review of the manuscript.

Santiago García: Approval of the final version of the manuscript; drafting and editing of the manuscript; intellectual participation in the propaedeutic and/or therapeutic conduct of the studied cases; critical review of the literature; critical review of the manuscript.

Ligia Aranibar: Approval of the final version of the manuscript; drafting and editing of the manuscript; intellectual participation in the propaedeutic and/or therapeutic conduct of the studied cases; critical review of the manuscript.

Fernando Valenzuela: Approval of the final version of the manuscript; drafting and editing of the manuscript; intellectual participation in the propaedeutic and/or therapeutic conduct of the studied cases; critical review of the literature; critical review of the manuscript.

## Conflicts of interest

None declared.
